# Epidemiology of gastrointestinal symptoms in young and middle-aged Swiss adults: prevalences and comorbidities in a longitudinal population cohort over 28 years

**DOI:** 10.1186/s12876-018-0749-3

**Published:** 2018-01-27

**Authors:** Maria Avramidou, Felix Angst, Jules Angst, André Aeschlimann, Wulf Rössler, Ulrich Schnyder

**Affiliations:** 1 Research Department, Rehabilitation Clinic (“RehaClinic”), Quellenstrasse 34, 5330 Bad Zurzach, Switzerland; 2 Department of Psychiatry, Psychotherapy and Psychosomatics, Psychiatric Hospital, University of Zurich, Zurich, Switzerland; 3 Institute of Psychiatry, Laboratory of Neuroscience (LIM 27), University of São Paulo, São Paulo, Brazil; 4 Department of Psychiatry and Psychotherapy, Charité University Medicine, Berlin, Germany; 5 Department of Psychiatry und Psychotherapy, University Hospital Zurich, University of Zurich, Zurich, Switzerland

**Keywords:** Epidemiology, Prevalence, General population survey, Associations, Odds ratios, Gastrointestinal, Functional

## Abstract

**Background:**

Although subacute and chronic gastrointestinal symptoms are very common in primary care, epidemiological date are sparse. The aim of the study was to examine and quantify the prevalence of subacute and chronic gastrointestinal symptoms and their associations with somatic and mental disorders in the general population.

**Methods:**

Data were collected prospectively between 1981 (age m = 22, f = 23) and 2008 (age 49/50) from the Zurich Cohort Study (*n* = 292 men, 299 women), a representative general population survey. The participants were assessed using a semi-structured interview, the “Structured Psychopathological Interview and Rating of the Social Consequences of Psychological Disturbances for Epidemiology” (SPIKE). Prevalence rates were computed to be representative of the general population aged 22–50. Associations were quantified by odds ratios (ORs) and their 99% confidence intervals (CI).

**Results:**

The prevalences of intestinal and of gastric symptoms were significantly higher among women in all categories examined. For example, any gastric symptoms: f. 26.4% vs m.15.2%; any intestinal symptoms: 27.6% vs 14.6%; nausea/vomitus: 19.1% vs 4.5%; constipation: 15.8% vs 6.5% (all *p* < 0.001). Strong associations (all *p* < 0.0001) were found between fatigue (1 month) and chronic stomach (OR = 9.96, 99%-CI: 5.53–17.94) and chronic intestinal symptoms (OR = 9.02, 99%-CI: 4.92–16.54). Panic attacks were associated with subacute intestinal symptoms (OR = 4.00, 99%-CI: 2.43–6.59). Anxiety was more strongly associated with subacute intestinal symptoms (OR = 3.37, 99%-CI: 2.23–5.08) than with subacute stomach symptoms (OR = 1.85, 1.20–2.86). Bipolar disorders were associated with subacute stomach symptoms (OR = 1.83, 1.18–2.17) and unipolar depression with subacute intestinal symptoms (OR = 2.05, 1.34–3.15).

**Conclusions:**

Remarkably high prevalence rates of gastric and intestinal complaints were observed in women (over 1/4; men 1/7). Fatigue/neurasthenia was the strongest co-factor in both conditions. Various syndromes related to anxiety, phobia, and panic disorders showed further significant associations. The integration of psychiatric and/or psychological treatment could help address the functional part of gastric and intestinal syndromes.

## Background

Gastrointestinal (GI) symptoms are widespread and carry heavy economic and social consequences. It is estimated that in the United States 11% of the population suffer from a chronic digestive disease, with a prevalence rate as high as 35% for those 65 years and over [1]. In 2010 alone, a reported 60 to 70 million people in the United States suffered from a digestive disease [2]. Although subacute and chronic gastrointestinal symptoms are very common, there has so far been no thorough investigation of the relevant Swiss epidemiological data, including the possible associations of gastrointestinal symptoms with somatic and psychiatric disorders in the general population.

Functional gastrointestinal disorders (FGIDs), represented by functional dyspepsia (FD) and irritable bowel syndrome (IBS), include variable combinations of chronic or acute gastrointestinal symptoms not explained by structural or biochemical abnormalities [3–5]. Worldwide the prevalence rates in the general population of dyspepsia/FD and IBS according to Rome III diagnostic criteria are 5.3–20.4% and 1.1–29.2%, respectively. Recent reports on Rome III FD and IBS indicate a female predominance [6, 7]. The prevalence of IBS subtypes is characterised by a male predominance for IBS with diarrhoea, and a female predominance for IBS with constipation. Genetic and environmental factors play an important role in the development of FGIDs. Gene polymorphisms are involved in the development of FGIDs. The prevalence of FGIDs differs across races and geographical areas. Food may also affect their development, but the causal relationships are not conclusive [8].

Psychosomatic concepts have long been postulated to account for irritable bowel syndrome in the absence of other objective aetiology and biomarkers, and it is common knowledge that many chronic conditions are associated with psychological disorders. It is worth noting that ulcerative colitis was interpreted by psychosomatic theory until the discovery of immune dysregulation [9].

In general, causal relationships are hard to establish. Clinical and population-based studies worldwide have found that some types of somatoform disorders (e.g., somatisation disorder, somatic-symptom-index (SSI) 4.6, and pain disorder) frequently co-occur with anxiety and depressive disorders. These findings could suggest either a causal relationship between those disorders or that they share certain aetiological factors. Irritable bowel syndrome occurs most frequently in young adults in response to emotional and other factors [10].

The aim of our analysis was to quantify the prevalence of gastrointestinal symptoms in Swiss adults between the ages of 22 (m)/23 (f) and 49/50 years, i.e. over a 28-year period, stratified by duration into subacute (1 week) and chronic (3 months, diarrhoea: 1 month). In addition, their associations with a range of somatic and psychiatric disorders were examined (see Methods section below).

Based on the current literature [7], our first hypothesis was that gastric and intestinal symptoms, especially chronic symptoms, are more prevalent in women than in men. Our second hypothesis was that there is a statistically significant association between gastrointestinal symptoms and certain psychiatric disorders, especially anxiety, suggesting that some GI symptoms are partially non-organic in nature.

## Methods

### Setting and data sampling

This study analyses data from the Zurich Study, an age cohort representative of the general population of the canton of Zurich, which accounts for approximately one sixth of the total Swiss population. The design was prospective, longitudinal, long-term, and naturalistic. The Zurich Study comprised a cohort of 4547 subjects (2201 men, 2346 women) representative of the canton of Zurich in Switzerland, who were screened in 1978 with the Symptom Checklist (SCL-90R), when the men were 19 and the women 20 years old. Male and female participants were sampled using different approaches. In Switzerland every male person undergoes military screening at the age of 19. With the consent of the military authorities but independently of their procedure, 50% of all male conscripts on the recruiting lists were randomly screened. Of these, 99.7% were included (*n* = 2201) (refusal rate 0.3%). The female sample was based on the complete population registers (civil status registers) of the canton of Zurich, from which 50% of the women born in 1958 were randomly selected. They were mailed questionnaires, to which *n* = 2346 (75%) responded. Because of the lower response rate of women with poorer educational levels, the interview sample was corrected for this bias. Written informed consent was obtained from all participants.

For economic reasons and in the interest of the long-term design of the study, in the second stage, a stratified sample of 591 subjects (292 men, 299 women) was selected for interview. The interview sample was enriched with persons reporting high levels of psychopathological symptoms and distress and therefore at risk for the development of psychiatric syndromes. Thus two thirds (*n* = 394) of the final interview sample consisted of randomly selected “high scorers” (defined by the 85th percentile or more on the Global Severity Index (GSI) of the SCL-90-R) and the remaining third (*n* = 197) of low scorers (<85th percentile). A detailed description of the Zurich Study design and method was published previously [11].

The use of such a two-phase method, consisting of initial screening and subsequent interview with a subsample stratified to enrich the sample with individuals of specific interest, was recommended in epidemiology [12]. Altogether, seven interview waves were carried out, when the participants were aged 20(m)/21(f) to 49/50: in 1979 (*n* = 591), 1981 (*n* = 456), 1986 (*n* = 457), 1988 (*n* = 424), 1993 (*n* = 407), 1999 (*n* = 367), and 2008 (*n* = 335, reflecting a 56.9% retention rate). Details of the interview waves and the drop-out rates and characteristics are published elsewhere [11]. The initial ratio of high-scorers to low-scorers (two thirds: one third) did not change significantly over the 30 years of the study; the sample therefore remained representative of the general population. The interview sample during the period 1981–2008 comprised 490 participants, who, weighted for stratified sampling, represent 2342 subjects (1141 men, 1201 women).

Data from the year 1979 were not included in the current analysis, because at that interview the assessment of symptom duration was limited to 1 month. In all subsequent interview waves a symptom duration up to 12 months was assessed, thus allowing the definition of chronic gastrointestinal problems (see below). We accordingly included the 490 subjects who took part in the interviews between 1981 and 2008. For each interview, all members of the initial sample of 1979 (*n* = 591) were followed up. In order to be included in our analysis, a participant had to have been interviewed at least once between 1981 and 2008. Because of the varying response rates the number of participants happened to be slightly higher in 1986 (*n* = 457) than in 1981 (*n* = 456).

### Instruments and measures

The instruments used comprised the Structured Psychopathological Interview and Rating of the Social Consequences of Psychological Disturbances for Epidemiology (SPIKE) [13], a clinical syndrome list (SL), the revised 90-item symptom check list (SCL-90R), a life-event inventory, and scales measuring coping behaviour and dissimulation. The interviews were conducted in the subjects’ homes by specially trained psychologists or psychiatrists and focused mainly on the past year in order to minimise forgetting.

The SPIKE is a semi-structured interview specifically developed for epidemiological surveys in psychiatric research. It assesses socio-demography, psychopathology, substance use, medication, health service use, impairment, and social activity. Data on its reliability and validity have been published previously [14]. Unlike other diagnostic interviews, which apply DSM (diagnostic and statistical manual) diagnoses using a top-down approach with multiple cut-offs, the SPIKE uses a bottom-up approach, assessing the past-year presence of about 14 somatic (including gastrointestinal) and 15 psychiatric syndromes, for each of which it checks symptoms, duration, frequency of episodes, distress, impairment and treatment.

The interviewers collected data on a total of 26 comorbid conditions: repeated panic attacks, anxiety (≥1 month Generalised Anxiety Disorder (GAD)), major depression (unipolar depression), bipolar depression, simple phobia, specific phobia, social phobia, agoraphobia, obsessive-compulsive disorder, suicidality, drug dependence, sedative/ tranquilliser dependence, alcohol dependence, smoking, bulimia, obesity, fatigue, sexual problems, sleep disorders, hypertension, hypotension, back pain, headache, other pain (not classified), cardiovascular symptoms and asthma spectrum conditions.

Interviewees were asked about specific gastrointestinal symptoms, for example: pain in the stomach, burning in the stomach, pressure in the stomach, nausea, vomitus, constipation, diarrhoea, intestinal pain, bloating, blood in the stool. Assessment on this pure symptom level provides obvious face and content validity. We defined as stomach symptoms, those symptoms perceived in the upper abdominal region (pain, burn, pressure, nausea, vomitus). Intestinal symptoms were defined as those located in the lower abdominal region (constipation, diarrhoea, pain, pressure, bloating). Participants were only asked about their symptoms; the cause of the symptoms was not evaluated. Consequently, liver and pancreas disorders could also cause gastrointestinal symptoms of the upper and lower gastrointestinal tract.

Participants were also specifically questioned about the duration and the intensity of their symptoms, in particular to specify the number of days they had experienced gastrointestinal symptoms over the past 12 months [11]. We defined as subacute, gastrointestinal symptoms lasting more than 1 week but less than 3 months: 1 week<= subacute<=3 months. Chronic symptoms that lasted 3 months or more: chronic≥3 months. An exception was made for diarrhoea: 1 week≤subacute< 1 month and chronic, which was defined as subacute if lasting 1 week or more but less than 1 month and as chronic ≥1 month. Acute gastrointestinal symptoms (< 1 week = 7 days) were excluded, because of the high probability that they were caused by a viral or bacterial infection. The intensity of the gastrointestinal symptoms/suffering experienced was subjectively quantified on a 0–100 mm “thermometer” scale: 0 = no distress/symptoms, 100 = maximum distress/symptoms [15]. The degree of distress/subjective suffering associated with a condition is an individual’s most important measure of their state of health and the primary reason for seeking help.

The main outcome was measured on the symptom level, for example, nausea, pressure, constipation, etc. The concomitant comorbidities were classified on the syndrome/disorder level, for example, major depression, specific phobia, asthma, etc.

### Analysis

The analysis of prevalence was limited to the period 1981–2008. All participants in at least one interview were included and analysed by weighting for stratified sampling. Descriptive statistics consisted of means (M) and standard deviations (SD) if the frequency distribution was approximately symmetrical, and of medians and quartiles (q1 and q3) if it was not. All data on prevalence were weighted back to the composition of the original, representative, population-based age cohort, comprising women born in 1958 and men born in 1959 living in the canton Zurich; quantification was in percentages (%) [13, 14].

Associations of gastrointestinal symptoms (present/absent as dependent variable) with psychiatric conditions were analysed by stepwise multivariate logistic regression to various comorbid conditions as independent variables, adjusting for sex and education level (confounding co-variates) in the interview sample of *n* = 490. We used backward stepwise regression, i.e., after each step the least significant variable was excluded. The observation period of the Zurich age cohort ended in 2008; thus associations were not confounded by age. The associations were expressed as odds ratios (ORs) with corresponding intervals of 99% confidence (99% CI). A type I error of 1% was chosen because the logistic models were stepwise determined after a maximum of 5 steps, which leads to a Bonferroni correction of 0.05/5. The OR quantifies the relative frequency (often also termed “risk”) of gastrointestinal symptoms if a given characteristic is present or not. All analyses were performed using SAS 9.3 for Windows (SAS Inc., Cary, NC, USA).

## Results

### Prevalences

Over the period 1981–2008 prevalence rates for any (subacute and chronic) stomach symptoms was 20.9% and for any intestinal symptoms 21.3%. (Tables 1 and 2).

**Table 1:** Numbers and weighted prevalence rates (%) of stomach symptoms by gender

		*N*	M + F%	M%	F%	*p*
Pain, burn, pressure	subacute≥1 week	65	9.3	7.9	10.8	0.012
	chronic≥3 months	51	8.8	4.1	13.2	< 0.001
	subacute+chronic	116	18.1	12.0	24.0	< 0.001
Nausea, vomitus	subacute≥1 week	36	7.0	4.8	9.0	< 0.001
	chronic≥3 months	40	5.5	0.6	10.1	< 0.001
	subacute+chronic	76	11.0	4.5	19.1	< 0.001
Pain, burn, pressure with nausea, vomitus	subacute≥1 week	35	4.9	2.9	7.1	< 0.001
	chronic≥3 months	33	5.0	0.6	9.0	< 0.001
	subacute+chronic	68	9.9	3.5	16.1	< 0.001
Any symptoms	subacute≥1 week	54	11.6	11.0	12.2	0.385
	chronic≥3 months	76	9.3	4.2	14.2	< 0.001
	subacute+chronic	130	20.9	15.2	26.4	< 0.001

Legend: *n* = number in the sample, prevalences: rates, back-weighted to the general population, M = male, F = female, *p* = type I error that the prevalences of m and f are different (two-sided)

**Table 2:** Numbers and weighted prevalence rates (%) of intestinal symptoms by gender

		*N*	M + F%	M%	F%	*p*
Constipation	subacute≥1 week	39	5.6	3.4	7.7	< 0.001
	chronic≥3 months	51	5.7	3.1	8.1	< 0.001
	subacute+chronic	90	11.3	6.5	15.8	< 0.001
Diarrhoea	subacute≥1 week	42	4.9	4.2	5.5	0.133
	chronic≥1 month	57	7.7	6.7	8.7	0.068
	subacute+chronic	99	12.6	10.9	14.2	0.013
Pain, pressure, bloating	subacute≥1 week	47	8.6	5.7	11.3	< 0.001
	chronic≥3 months	66	8.1	4.6	11.4	< 0.001
	subacute+chronic	113	16.7	10.4	22.7	< 0.001
Any symptoms	subacute≥1 week	57	9.0	6.3	11.7	< 0.001
	chronic≥1 or 3 months	91	12.2	8.3	16.0	< 0.001
	subacute+chronic	148	21.3	14.6	27.6	< 0.001

Legend: *n* = number in the sample, prevalences: rates, back-weighted to the general population, M = male, F = female, *p* = type I error that the prevalences of m and f are different (two-sided)

Among the gastric symptoms (Table 1), overall pain/burn/pressure was more common (18.1%) than nausea/vomitus (11.0%). For subacute stomach pain, burn (reflux), pressure the prevalence rate was 10.8% in women and 7.9% in men. The rates by gender for the corresponding chronic symptoms were 13.2 and 4.1%. Indeed, the prevalence rates for all examined symptom groups were consistently higher in women, with the differences being statistically highly significant except in the subgroup of any subacute symptoms. The widest gender difference was 10.1% vs 0.6% for chronic nausea, vomitus. Overall, there was a female preponderance (26.4% vs 15.2%) for any (subacute + chronic) gastric/stomach symptoms.

The prevalence rates in every group of intestinal symptoms examined were also higher in women (Table 2). With the exception of diarrhoea, the gender differences were statistically highly significant: constipation subacute 7.7% vs 3.4%, chronic 8.1% vs 3.1%; pain/pressure/bloating subacute 11.3% vs 5.7%, chronic 11.4% vs 4.6%. Overall, the female preponderance was 27.6% to 14.6% for any (subacute + chronic) intestinal symptoms. Pain/pressure/bloating was the most prevalent intestinal syndrome (16.7%).

### Distress (scale 0–100)

The highest distress levels were found in 54 subjects with chronic gastric symptoms, with a mean of 67.9 (standard deviation: 24.0), followed by 76 subjects with subacute gastric symptoms with a mean of 55.4 (standard deviation: 26.3). There were no significant distress-related gender differences (Table 3).

**Table 3:** Distress levels of any symptoms (0 = no to 100 = maximum distress)

Gastric	*N*	mean	sd	Intestinal	*n*	mean	sd
subacute all	76	55.4	26.3	subacute all	57	50.9	28.1
subacute m	33	52.5	26.5	subacute m	20	46.0	26.4
subacute f	43	57.6	26.2	subacute f	37	53.5	28.9
p (m vs f)		0.399				0.330	
chronic all	54	67.9	24.0	chronic all	91	51.9	30.3
chronic m	17	69.3	23.9	chronic m	33	50.5	32.8
chronic f	37	67.3	24.3	chronic f	58	52.7	29.1
p (m vs f)		0.776				0.733	

Legend: *n* = number in the sample, m = male, f = female, mean = arithmetic mean, sd = standard deviation, *p* = type I error that the distress of m and f are different (two-sided)

### Comorbid conditions

Stepwise logistic regression modelling selected all conditions having significant associations with gastrointestinal symptoms, adjusting for sex and level of education; no adjustment for age was necessary, since all interviewees had the same age per assessment time point.

**Fig. 1:**
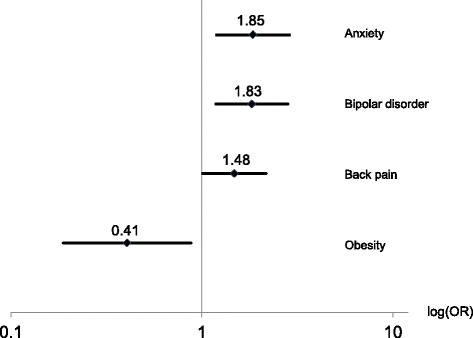
Associations with subacute stomach symptoms (*n* = 76 with, *n* = 410 without symptoms): Odds ratios (OR), 99% CI, adjusted for sex and education level. Explained variance: 15.7%. Legend: Odds ratios (OR), 99% CI, adjusted for sex and education level

Subacute stomach symptoms (Fig. 1) were found to be moderately associated with anxiety (OR = 1.85, 99%-CI: 1.20–2.86), bipolar disorder (1.83, 1.18–2.82) and back pain (1.48, 1.01–2.17). An inverse (protective) association was found between subacute stomach symptoms and obesity (0.41, 0.19–0.88).

**Fig. 2:**
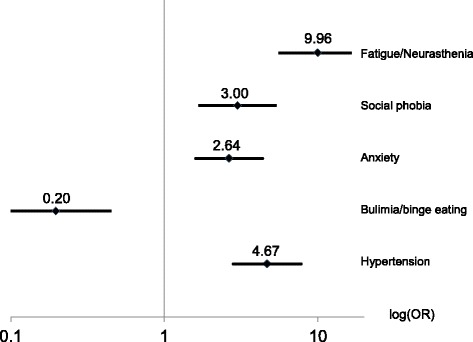
Associations with chronic stomach symptoms (*n* = 54 with, *n* = 372 without symptoms): Odds ratios (OR), 99% CI, adjusted for sex and education level. Explained variance: 52.4%. Legend: Odds ratios (OR), 99% CI, adjusted for sex and education level

Chronic stomach symptoms (Fig. 2) were strongly associated with fatigue (OR = 9.96, 99%-CI: 5.53–17.94) and moderately with high blood pressure (4.67, 2.80–7.78), social phobia (3.00, 1.70–5.27) and anxiety (2.64, 1.60–4.36). An inverse association was found with bulimia/binge eating (0.20, 0.09–0.45).

**Fig. 3:**
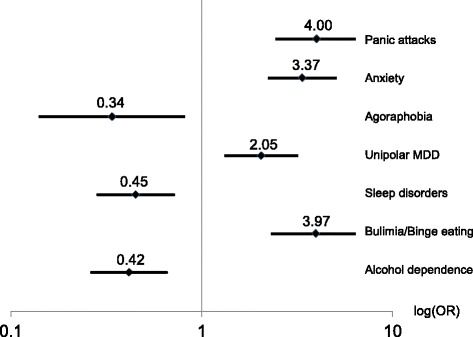
Associations with subacute intestinal symptoms (*n* = 78 with, *n* = 345 without symptoms): Odds ratios (OR), 99% CI, adjusted for sex and education level. Explained variance: 38.2%. Legend: Odds ratios (OR), 99% CI, adjusted for sex and education level

Subacute intestinal symptoms (Fig. 3) were strongly associated with panic attacks (OR = 4.00, 99%-CI: 2.43–6.59), bulimia/binge eating (3.97, 2.30–6.82), anxiety (3.37, 2.23–5.08) and moderately with unipolar major depression (2.05, 1.34–3.15). Inverse associations were documented for sleep disorders (0.45, 0.29–0.71), alcohol dependence (0.42, 0.27–0.65) and agoraphobia (0.34, 0.14–0.82).

**Fig. 4:**
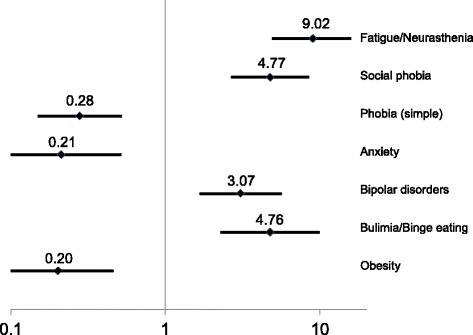
Associations with chronic intestinal symptoms (n = 76 with, n = 345 without symptoms): Odds ratios (OR), 99% CI, adjusted for sex and education level. Explained variance: 45.7%. Legend: Odds ratios (OR), 99% CI, adjusted for sex and education level

Chronic intestinal symptoms (Fig. 4) were very strongly associated with fatigue (OR = 9.02, 99%-CI: 4.92–16.54), social phobia (4.77, 2.66–8.54) and bulimia (4.76). A moderate association was found with bipolar disorder (3.07, 1.69–5.57). Inverse (protective) associations were documented for obesity (0.20, 0.09–0.46), specific/simple phobia (0.28, 0.15–0.52) and anxiety (0.21, 0.09–0.52).

## Discussion

This study provides new insights into the longitudinal epidemiology of functional gastrointestinal symptoms in Switzerland. In our sample of 490 participants, male and female, one in five complained of subacute or chronic stomach symptoms and of subacute or chronic intestinal symptoms. Stomach symptoms were defined as symptoms of the upper gastrointestinal tract (pain, burn, pressure, nausea, vomitus) and intestinal symptoms as symptoms of the lower gastrointestinal tract (constipation, diarrhoea, pain, pressure, bloating). Pain and pressure were most prevalent among the gastric as well the intestinal symptom groups. For both sexes, the prevalences of subacute and chronic complaints were comparable in most of the symptom groups.

In a review of the prevalence of functional dyspepsia, two studies could be found that were comparable to ours [16]. A British study of a randomly selected sample of 20% of patients registered at two health centre practices who were examined by endoscopy to exclude organic disease reported an estimated prevalence of functional gastrointestinal symptoms of 11.5%. A carefully designed endoscopy study of the inhabitants of a Norwegian community (Sørreisa) aged 20–69 found a prevalence of functional gastrointestinal disorders of 14.7%. Comparing those results (11.5% and 14.7%) to our own prevalence rates of around 20% of any gastric symptoms, it may be concluded that 1/2 to 3/4 of the gastric symptoms are functional in our sample.

Our analysis focused on gastrointestinal (GI) symptoms of at least 1 week’s duration, which excludes acute infections but covers most of the functional syndromes. People with functional GIs suffer by definition from disorders without somatic background, which can be very difficult to understand and to accept. The symptoms, which cause shame and embarrassment, tend to isolate the sufferer and impact heavily on their working and social lives. It may thus be assumed that there is a bidirectional relationship between functional GI symptoms and psychiatric disorders, with the latter being both partial cause and frequent product of the former, creating a vicious circle.

We found the highest distress levels in subjects with chronic followed by subacute gastric symptoms. The correlation between chronic symptoms and higher distress levels might be explained by exhaustion (fatigue/neurasthenic syndrome) as discussed later. Women and men were equally distressed by subacute and chronic gastrointestinal symptoms although more women had GI illnesses.

As initially hypothesised, our study found higher female prevalence rates in all subgroups of gastrointestinal syndromes examined. Our findings are in line with earlier studies. In Smith’s epidemiological study of 1978 women with IBS outnumbered men by a ratio of 2.3:1 [1]. However, a recent Japanese report showed a male predominance for the IBS-subtype with diarrhoea and a female predominance for the IBS-subtype with constipation, the overall prevalence of IBS being assessed at 13.1%, 15.5% for females and 10.7% for males [8].

In our study the strongest associations overall were found between fatigue and chronic stomach and chronic intestinal symptoms. This finding is compatible with the results of a recent study showing a strong association between fatigue and depression in patients with Crohn’s disease and ulcerative colitis [17]. This raises the perennial question of “cause or effect”, as fatigue could be an important aetiological factor in the development of functional gastrointestinal symptoms but also the result of those symptoms. The key role of fatigue in chronic gastrointestinal symptoms needs further research.

Hypertension was relatively strongly associated with chronic stomach symptoms. Hypertension could be a primary or secondary manifestation of rhythm disturbances or angina, which are thought to be associated with gastrointestinal symptoms. This hypothesis is supported by an interesting review, which found associations between rhythm disturbances, linked angina pectoris and oesophageal diseases [18]. Further, inflammatory large bowel disorders, were associated with hypotension and hypertension.

We found a moderate association between back pain and subacute stomach symptoms. Interestingly, obesity was inversely correlated with subacute stomach and chronic intestinal symptoms. Or rather, conversely, it is possible that people who do not suffer from stomach or intestinal problems are more prone to obesity than those who do; this may be due to the fact that gastrointestinal problems limit food intake. Bulimia and binge eating, however, had a fourfold higher association with subacute and chronic intestinal symptoms but an inverse association with chronic stomach symptoms. To the best of our knowledge there is no literature that elucidates these relationships.

In line with our initial hypothesis that GI symptoms correlate with psychiatric disorders, we found that some anxiety states (social phobia, anxiety ≥1 month Generalised Anxiety Disorder – GAD) tended to be associated to varying degrees with every subgroup of gastrointestinal disorders (Figs. 1, 2, 3 and 4). An exception was specific phobia, which was negatively associated with chronic intestinal symptoms.

In our study major mood disorders were also varyingly associated with GI symptoms: bipolar disorder with subacute stomach and chronic intestinal symptoms, and major depression with subacute intestinal symptoms but not with the chronic forms.

There is ample literature on the role of emotional factors in functional gastrointestinal symptoms. An early study by Young et al. reported a “psychiatric illness” in 72% of a group of participants with irritable bowel syndrome (IBS), compared to 18% in the control group [19]. The prevalence of depression and anxiety in patients with IBS and ulcerative colitis (UC) has been reported in three studies. Hartono et al. found an excess prevalence of depression and anxiety of 38.7% and 6.5% respectively in IBS patients relative to healthy patients [20]. Uz et al. reported an excess prevalence of depression or anxiety of 34% and 2% in patients with IBS in comparison to healthy controls [21]. Shah et al. conducted a systematic review on prevalence rates of anxiety and depression in patients with IBS and with UC. In addition to the above-mentioned prevalence rates for anxiety and depression in IBS patients, the authors documented an excess prevalence of anxiety in UC patients relative to healthy controls of 41.7% (22.2% baseline prevalence in healthy controls) based on a State-Trait Anxiety Inventory (STAI) score greater than 40. Excess depression of 38.9%, with a baseline prevalence of 11.1% in the healthy population, was evaluated with a Zung Depression Scale (ZDS) score greater than 49. The authors concluded that both IBS and UC are associated with psychological disorders versus healthy controls and that the association between irritable bowel syndrome and psychological factors might be attributed to the psychological suffering caused by this chronic and debilitating disease [9].

Further studies have confirmed associations between GI symptoms and syndromes and anxiety and depression. A Canadian study of 2015 showed an association between irritable bowel syndrome and general anxiety [22]. Other recent studies have reported an association between gastrointestinal reflux disease and visceral anxiety [23]. An American study, which focused on correlations of anxiety disorders and physical health conditions in elderly Americans, found a statistically significant association between certain anxiety forms and gastrointestinal disease [24]. A study of 2016 showed a clear association of gastrointestinal reflux disease with anxiety and depression in Australian men [25].

Our findings are compatible with the above studies as regards a significant association between gastrointestinal symptoms and anxiety. However, the association with depressive syndromes in our data was limited to subacute intestinal symptoms only.

The significant associations found between GIs and other somatic and psychiatric disorders, could suggest the need to modify the clinical approach and treatment in the field. In particular, the evaluation and treatment of patients with gastrointestinal symptoms would benefit from the integration of psychiatric and/or psychological aspects, broadening the perspective on the pathophysiological mechanisms involved.

The most important strengths of the Zurich Cohort Study are its long-term longitudinal and comprehensive design covering all relevant psychiatric and somatic issues. The study greatly benefited from the continued large number of participants and from the multiple follow-ups conducted over decades. The multivariate logistic models attained high variances, i.e. explained a large proportion of the characteristics of gastrointestinal symptoms, indicating that the assessment of the determining factors was comprehensive. Furthermore, the logistic regression models were adjusted for sex and level of education, which were both kept in the model irrespective of their significance and which are well-known important confounders.

The study also has limitations: by virtue of its longitudinal design the participation rate fluctuated across the six interviews (1981–2008). Furthermore, the associations were examined over the whole observation period and no time-linked cause-effect relationships were analysed. No causal conclusions can therefore be drawn from our association data. Finally all data are interview based without physical examination.

## Conclusions

This study provides new insights into the epidemiology of gastric and intestinal symptoms in young and middle-aged persons in the general population.

Functional gastric and intestinal symptoms are remarkably common in the general population, especially in women. We found very strong associations between fatigue/neurasthenia and both conditions and significant associations with various somatic and mental (especially anxiety-related) syndromes. Since there can be no early clarification of the “cause or effect” relationship between GI symptoms and accompanying psychiatric syndromes, screening of functional GI patients followed by the integration of appropriate psychiatric and/or psychological treatment could be an important tool in addressing effectively the functional part of their condition.

More research is needed to clarify the role of fatigue, anxiety disorders and major mood disorders as primary cause or secondary expression of gastrointestinal symptoms.

## Abbreviations

CI: Confidence interval
DSM: Diagnostic and Statistical Manual
FD: Functional dyspepsia
FDIG: Functional gastrointestinal disorder
GAD: Generalised anxiety disorder
GI: Gastrointestinal
GSI: Global Severity Index
IBS: Irritable bowel syndrome
M: Mean
OR: Odds ratio
SCL-90R: Revised 90-item Symptom Check List
SD: Standard deviations
SL: Syndrome list
SPIKE: Structured Psychopathological Interview and Rating of the Social Consequences of Psychological Disturbances for Epidemiology
SSI: Somatic-Symptom-Index
STAI: State-Trait Anxiety Inventory
UC: Ulcerative colitis
ZDS: Zung Depression Scale
